# Validation of turbulent kinetic energy in an aortic coarctation before and after intervention - MRI vs. CFD

**DOI:** 10.1186/1532-429X-15-S1-E46

**Published:** 2013-01-30

**Authors:** Jonas Lantz, Tino Ebbers, Jan E Engvall, Matts Karlsson

**Affiliations:** 1 Department of Management and Engineering, Linköping University, Linköping, Sweden; 2 Department of Medical and Health Sciences, Linköping University, Linköping, Sweden; 3 Center for Medical Image Science and Visualization, Linköping University, Linköping, Sweden

## Background

The blood flow through aortic coarctations can undergo transition to turbulence, as the narrowing causes the velocity to increase. Increased levels of turbulent kinetic energy (TKE) are undesirable, as energy is drawn from the mean flow to the turbulent fluctuations. It has recently been shown that TKE in blood flow can be measured with MRI [1], and the aim of this study is to assess the turbulent kinetic energy before and after catheter intervention. Simulations of the flow were performed using computational fluid dynamics (CFD) and compared with MRI measurements.

## Methods

A 63 year old female with an aortic coarctation was examined in a 1.5T Philips Achieva MRI system before and after balloon dilatation. The catheter intervention resulted in a decrease in pressure drop over the constriction and an increased flow rate. Measurements of the velocity and turbulent kinetic energy in the whole aorta over the complete cardiac cycle were obtained using navigator-gated time-resolved 3D phase-contrast MRI [1]. Two scans with different motion encoding strengths, 2.0 and 3.5 m/s, were obtained to acquire TKE and velocity data, respectively [2]. Blood flow in the aorta was also obtained using CFD, with boundary conditions obtained from contrast enhanced MRA and 2D through-plane velocity measurement in the ascending aorta. The flow simulations employed an advanced turbulence model (Large Eddy Simulation, LES) to resolve the turbulent flow scales. Results were extracted along the geometrical centerline of the aorta and plotted in a spatiotemporal manner, enabling an investigation of TKE both along the aorta and throughout the cardiac cycle.

## Results

TKE is an estimator of the amount of turbulence present in the flow. In the pre-intervention case, both CFD and MRI showed large values of TKE at two locations in the aorta; in the throat of the coarctation and in the aortic arch where a sharp bend of the aorta was present. After intervention, total TKE levels decreased even though the cardiac output increased. This can be explained by the larger cross-sectional area after intervention, effectively reducing local velocity. MRI and CFD results both showed that turbulent flows were present from the aortic arch to the lower descending aorta during systole, and then dissipated during the beginning of diastole. Peak values of TKE occurred during systolic deceleration and not at peak flow rate, as, in general, accelerating flows tend to have a stabilizing effect, while decelerating flows are more prone to break up.

## Conclusions

The agreement between CFD and MRI results was very good, with both methods predicting transition to turbulence at the same locations in the aorta and with the same TKE magnitudes. As the methods and data used to obtain TKE by MRI and CFD are completely different, these results confirm that MRI-measured TKE is reliable and accurate.

## Funding

Swedish Research Council, the Swedish Heart-Lung Foundation

**Figure 1:**
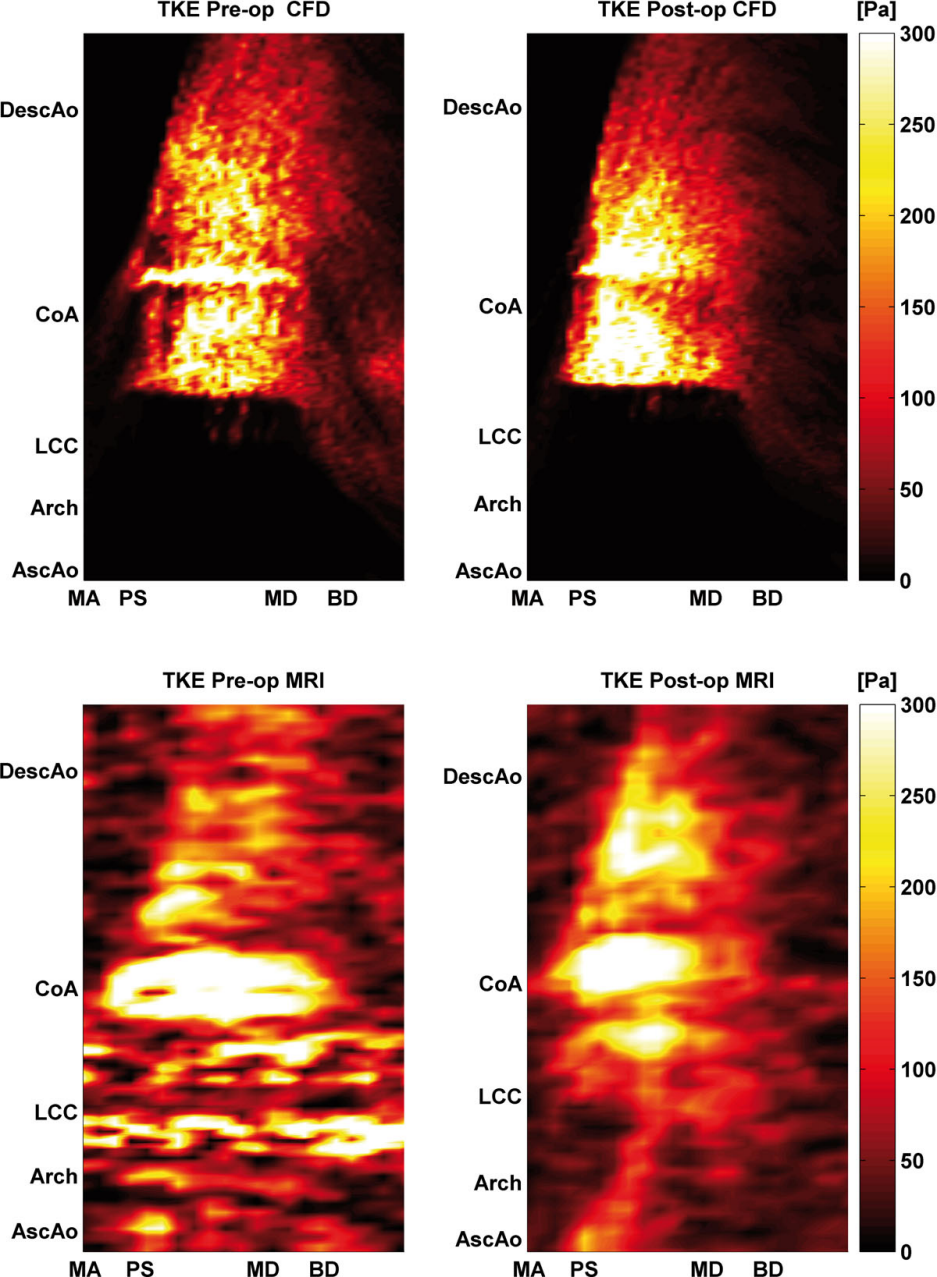
TKE along the centerline as a function of time in the pre- and post-intervention cases; comparison between CFD (top row) and MRI (lower row). X-axis labels: MA: maximum acceleration, PS: peak systole, MD: maximum deceleration, BD: beginning of diastole. Y-axis locations are AscAo: ascending aorta, Arch: aortic arch, LCC: left common carotid artery, CoA: coarctation of the aorta, and DescAo: descending aorta.
